# Biomarkers can predict potential clinical responders to DIMS0150 a toll-like receptor 9 agonist in ulcerative colitis patients

**DOI:** 10.1186/1471-230X-14-79

**Published:** 2014-04-23

**Authors:** Nikolai V Kuznetsov, Arezou Zargari, Alexander W Gielen, Oliver D von Stein, Eugen Musch, Ragnar Befrits, Robert Lofberg, Petra von Stein

**Affiliations:** 1 InDex Pharmaceuticals, Tomtebodavägen 23a, 171 77 Stockholm, Sweden; 2 Clinic of Colo-Proctology and Intestine Center, Marienhospital, Bottrop, Germany; 3 Gastroenterology and Hepatology Clinic, Karolinska University Hospital, Solna, Sweden; 4 Department of Medicine, Karolinska Institutet and Stockholm Gastro Center, Stockholm, Sweden

**Keywords:** Ulcerative colitis, Glucocorticosteroids, Steroid refractory, Biomarker, Companion diagnostics

## Abstract

**Background:**

Glucocorticoids (GCS) remain one of the mainstay treatments in the management of ulcerative colitis (UC) but up to a third of patients will ultimately fail to respond and progress to a more severe and difficult to manage disease state. Previous clinical studies suggest that the Toll-Like Receptor 9 (TLR9) agonist DIMS0150 not only induces production of key anti-inflammatory cytokines as IL-10 but interestingly also enhances steroid sensitivity in steroid refractory UC patients. We investigated, in the context of a clinical study, whether a pre-selection of steroid response genes could identify steroid refractory UC subjects most likely to respond to DIMS0150 treatment.

**Methods:**

In a non-interventional pilot study, blood from steroid refractory UC patients and healthy volunteers was taken and thirty-four previously described steroid response genes were analysed by real time PCR analysis. To establish clinical utility of the identified biomarkers, a placebo controlled, randomized, double blinded study in active steroid dependent and steroid resistant UC patients on concomitant steroid therapies was used (EudraCT number: 2006-001846-15).

**Results:**

We identified three potential biomarkers CD163, TSP-1 and IL-1RII whose response to steroids was significantly enhanced when DIMS0150 was applied. Thirty-four subjects were randomized to receive a single rectal administration of placebo or 30 mg of DIMS0150. Blood derived PBMCs were obtained prior to dosing and assayed for evidence of a steroid enhancing effect following steroid incubation in the presence of DIMS0150. Comparison to established steroid sensitivity marker IL-6 confirmed that clinical responders are steroid refractory UC patients. Upon study completion and un-blinding, the biomarker assay correctly predicted a clinical response in over 90% of the patients.

**Conclusion:**

Using specific steroid response biomarkers, GCS refractory UC patients most likely to benefit from DIMS0150 treatment could be identified and illustrates the usefulness of a personalized treatment approach.

## Background

Ulcerative colitis (UC) is a disease characterised by chronic inflammation of the rectal and colonic mucosa. The medical management of UC is divided into treatment of active disease and maintenance of remission. The treatment of patients with UC aims to reduce inflammation and promote colon healing and mucosal recovery. In the majority of cases the disease may be controlled with conventional drugs including sulphasalazine (SASP), 5-aminosalicylic acid (5-ASA) and glucocorticosteroids (GCS) [1,2]. GCS are one of the mainstay treatments in the management of inflammatory bowel disease (IBD). GCS are potent anti-inflammatory agents widely used for the suppression of inflammation in chronic diseases such as asthma, rheumatoid arthritis, IBD and autoimmune diseases [3]. However, 20-50% of IBD patients will fail to respond to steroid therapy [4] resulting often in a more difficult to manage disease course. Failure to respond to GCS therapy is an indication for potential surgical intervention although immunomodulatory agents such as intravenous cyclosporine or tumor necrosis factor (TNF) inhibitors have demonstrated some effectiveness at reducing colectomy rates on a short-term basis [5,6].

DNA based immunomodulatory sequence (DIMS0150) is a single stranded partially modified synthetic oligonucleotide of 19 bases in length. The drug functions as an immunomodulator by activating the Toll-Like Receptor 9 (TLR9) present in immune cells such as B-cells, macrophages and plasmacytoid dendritic cells (pDCs) that are found in abundance on mucosal surfaces such as the colonic mucosa. Through rectal administration of DIMS0150 in the form of an enema, the agent comes in direct contact with a large number of target cells thereby ensuring a robust immunomodulatory response. Activation of TLR9 by DIMS0150 results in the local production of potent anti-inflammatory cytokines such as IL-10 and type I interferons that have also interestingly been shown to increase steroid sensitivity in cells derived from steroid-resistant UC patients [7] and human monocytes [8].

Results from two previous clinical studies in UC patients treated with a single rectal dose of DIMS0150 have indicated that steroid refractory UC patients benefit from the treatment. In the first clinical study, a single dose of DIMS0150 was given to steroid unresponsive IBD patients on concomitant steroid therapies [9]. The study illustrated that both single dose levels used (3 mg and 30 mg) were effective in inducing a clinical response. After one week five out of seven patients (70%) that received active treatment had a clinical response and two have remained, after more than 8 years, in GCS free remission. One of four patients receiving placebo responded but in a transient manner.

A larger phase II study evaluated the ability of DIMS0150 at four dose levels (0.3, 3, 30 and 100 mg) administered as a single rectal dose to induce clinical remission in 151 patients with mild or moderately active UC compared to placebo. No significant benefit was demonstrated at any dose level suggesting that the lack of efficacy was possibly due to the different patient target group (data not published). The target groups of these studies differed in two ways, firstly the second trial was conducted in less severe UC patients and secondly concomitant steroid therapy was an exclusion criteria. To investigate whether the effect of DIMS0150 in the first study could be linked to steroid sensitivity, we screened 34 known steroid response genes in an *in vitro* assay. Additionally, to be able to re-affirm whether steroid refractory UC subjects on concomitant steroid therapies are the relevant target group or whether the combination of DIMS0150 and steroid therapy is needed, a phase IIa proof of concept (PoC) study was conducted in steroid dependent or steroid resistant UC patients on concomitant steroid therapies addressing DIMS0150 at a single dose level of 30 mg.

According to European Crohn’s and Colitis organisation (ECCO) guidelines, the definition of steroid resistance is a failure to respond to 0.75 mg/Kg body weight intravenous administered steroids given over 3 to 5 days [10]. Likewise, steroid dependency is defined as the inability to reduce steroid usage below 10 mg/day without recurrent active disease. We reasoned that to determine the clinical picture of steroid sensitivity by these means would greatly impact the rate of inclusion into the PoC study with many patients opting not to undergo these demanding procedures. Consequently, we employed the use interleukin 6 (IL-6) that has gained significant recognition as a suitable biomarker for determining the steroid sensitivity status of a subject in published research as well as its use in human clinical studies addressing steroid resistant disorders such as asthma and ulcerative colitis [11-14]. This PoC study also enabled us to evaluate the suitability of potential biomarkers for DIMS0150 whether they could be used to predict a clinical response.

The results from this PoC phase IIa study indicate that GCS refractory patients on concomitant GCS therapy respond more favourably to a single dose of DIMS0150 and the utility of the biomarkers CD163, TSP-1 and IL-6 in confirming the right target group and predicting a most likely response to DIMS0150 could be demonstrated. Using the biomarkers we could also demonstrate a steroid re-sensitizing effect *in vivo* following DIMS0150 treatment in a case report from a named patient basis program and confirm that after treatment with DIMS0150, GCS can be used to successfully treat a new disease flare in a patient.

## Methods

### Study design and patient population

#### *Identification of potential steroid response biomarkers*

A study including 9 steroid resistant active UC patients and 9 healthy volunteers (mean age of 44.7 and 44.3, female to male ratio of 2:7 and 6:3, 9 Caucasians and 8 Caucasians plus one Latin American, respectively) was performed to donate blood at one occasion in one study centre in Stockholm, Sweden.

#### *Proof of concept (PoC) phase IIa study (EudraCT number: 2006-001846-15)*

The study was placebo controlled, randomized, double-blinded and conducted in Sweden and Russia in 17 study centres. Steroid resistant or steroid dependent patients (Intention-To-Treat population ITT = 34) on a stable and tolerable steroid dose of at least 5 mg/day for 2 weeks with mild to moderate UC with DAI score [2] of 6 – 11 at screening visit were blindly allocated to active treatment (DIMS0150) or placebo in a 2:1 ratio ((ITT = 22) versus (ITT = 12) respectively). Concomitant stable treatment with 5-ASA or SASP was allowed during the study period but patients treated with anti-TNFα or cyclosporine were excluded from the study. The drug was given as a single 50 mL rectal enema consisting of 30 mg of DIMS0150 in sterile water or sterile water only in case of placebo. Primary objective of the study was to evaluate clinical response compared to placebo, secondary objectives included safety and tolerability as well as evaluation of biomarkers in comparison to the steroid sensitivity marker IL-6. Baseline characteristics of the patients are outlined in Table 1. The study started in January 2007 and was terminated early in April 2009 because it was judged that allowing all subjects to complete all scheduled visits would not provide additional meaningful data in lieu of the primary endpoint.

**Table 1:** Baseline characteristics of study population proof of concept phase IIa study

**Demographic data**		**DIMS0150 (n = 22)**	**Placebo (n = 12)**
Gender	Male (%)	12 (54.55)	6 (50.00)
	Female (%)	10 (45.45)	6 (50.00)
Race	Caucasian	22 (100.00)	12 (100.00)
Age (years)	Mean (SD)	41.3 (14.96)	39.7 (12.37)
	Min, Max	23.0, 72.0	22.0, 56.0
**Other characteristics**		**DIMS0150 (n = 22)**	**Placebo (n = 12)**
Disease duration (days)	n	16	7
	Missing	6	5
	Mean (SD)	916.1 (871.85)	1760.6 (806.71)
	(Min, Max)	(154.0, 3555.0)	(922.0, 3003.0)
DAI score at screening	n	22	12
	Missing	0	0
	Mean (SD)	7.9 (1.19)	7.9 (1.56)
	(Min, Max)	7.0, 10.0	6.0, 11.0
UC extent	Beyond splenic flexure	3 (13.64%)	2 (16.67%)
	Up to splenic flexure	10 (45.45%)	6 (50.00%)
	Up to sigmoid descending junction	7 (31.82%)	3 (25.00%)
	Up to recto-sigmoid junction	1 (4.55%)	1 (8.33%)
	Not known	1 (4.55%)	0 (0.00%)
Number of subjects taking glucocorticoids medication during the study		20 (90.91%)	11 (91.67%)
Corticoids acting locally	Number of steroid medications (PT)	3	
	Budenoside	2 (9.09%)	
	Prednisolone sodium phosphate	1 (4.55%)	
Glucocorticoids	Number of steroid medications (PT)	21	12
	Hydrocortisone	1 (4.55%)	1 (8.33%)
	Methylprednisolone	1 (4.55%)	1 (8.33%)
	Prednisolone	19 (86.36%)	10 (83.33%)

Steroids medication (PT) that occur more than once during the study period will be counted only once within a subject.

Percentages calculated for the number of subjects in the safety population by treatment group.

#### *Named patient-based treatment example*

One chronic active treatment refractory UC patient (male, 50 years, pancolitis, Caucasian) was treated three times with DIMS0150 with four weeks between dosing occasions [15].

### Study drug

DIMS0150 is a fully synthetic oligodeoxynucleotide with the sequence 5′-G*G*A*ACA GTT CGT CCA T*G*G*C-3′ where (*) indicates phosphorothioate linkages, produced by Avecia (Milford, USA) and prepared as study drug by Apoteksbolaget AB, Production & Laboratory, Umeå, Sweden. The randomisation code was produced by a computer-generated procedure, which used the method of randomly permuted blocks. Double-blinding against water was accomplished by giving all study products identical appearance, packaging and labelling.

### Blood collection and RNA isolation for IP-10 expression analysis

Whole blood (5 mL) from 26 patients of the PoC study (all patients included after Amendment 5 of original protocol) was collected in PAXtubes (PreAnalytiX, Hombrechtikon, Switzerland) just before drug administration and 4 hrs after. RNA isolation was performed according to the manufacturer’s guidelines using PAXgene Blood RNA Kit (Qiagen, Hilden, Germany).

### Blood collection and PBMC isolation and stimulation

From 32 patients of the PoC study, 30 mL blood was collected in sodium heparin tubes (Venoject®, Teruma Sweden AB) and peripheral blood mononuclear cells (PBMC) isolated 24 hrs after collection by density gradient centrifugation using Ficoll-Paque Plus (Pharmacia Biotech, Stockholm, Sweden). PBMCs were washed three times in buffered saline solution, and resuspended in complete RPMI 1640 medium (Sigma, St. Louis, USA).

PBMCs were seeded in 96 well plates (0.5 x 10^6^ cells/well), cells stimulated for 48 hrs with DIMS0150 (25 μM or 100 μM) in the presence or absence of Dexamethasone (10^-6^, 10^-8^, 10^-10^ M; Sigma). Cell supernatant was collected and kept at -20°C for cytokine analysis. The cells were covered with 50 μl/well of RLT-lysis buffer (Qiagen) containing 1% of β-mercaptoethanol and kept at -20°C for mRNA isolation.

### Cytokine analysis

IFN-γ, IL-6, IL-10, IP-10, and TNF-α were measured using cytometric bead array (CBA) flex kit (Becton Dickinson) according to the manufacturer’s instructions on a FACSArray flow cytometer using FCAP Array software (Becton Dickinson, New Jersey, USA). IFN-α was analyzed using human IFN-α Multi-subtype ELISA kit (PBL, Biomedical Laboratories, New Jersey, USA) and IFN-β was detected with human IFN-β ELISA kit (Fujirebio INC., Tokyo, Japan) according to the manufacturer’s instructions.

### Biomarker analysis

RNA isolation was performed using Qiagen RNeasy RNA isolation kit and Qiacube (Qiagen) according to manufacturer’s guidelines.

For first strand cDNA synthesis, 0.3-1.0 μg of total RNA/sample and 10pM of the Oligo-dT-primer (5′-dT_20_NV-3′) was taken. The reactions were performed using Superscript II according to the manufacture’s guidelines (Invitrogen, Carlsbad, USA).

Real-time PCR (qPCR) was performed on an ABI 7500 Real-Time PCR system using Power SYBR®Green PCR Kit according to the manufacture’s guidelines (Applied Biosystems). Reactions were performed in triplicates using 1 μl 1:10 diluted cDNA per reaction and qPCR data were analyzed using relative quantification via 2^-∆∆Ct^ method (SDS 1.3 software, Applied Biosystems, Foster City, USA) and γ-actin as internal control (Table 2). Gamma-actin was chosen because it showed consistent expression in stimulated and non-stimulated PBMCs and can be employed in the same cDNA dilution as the target genes.

**Table 2:** Primer information qPCR

**Gene**	**ID #**	**Forward primer sequence**	**Reverse primer sequence**
γ-actin	71	5′-TGCCGACAGGATGCAGAA-3′	5′-GGGTGCGATGATCTTGATCTTC-3′
IP-10	3627	5′-TGAAAAAGAAGGGTGAGAAGAGATG-3′	5′-TTTAGACCTTTCCTTGCTAACTGCTT-3′
CD163	9332	5′-GCTGCAGTGAATTGCACAGATAT-3′	5′-CGGGATGAGCGACCTGTT-3′
TSP-1	7057	5′-CGGGATGAGCGACCTGTT-3′	5′-GTACTGAACTCCGTTGTGATAGCATAG-3′
IL-1RII	7850	5′-TCACTAGGAGTATTGAGCTACGCATC-3′	5′-ATTGTCAGTCTTGACCCCAGAGA-3′
IL-6	3569	5′-AGCCCTGAGAAAGGAGACATGTA-3′	5′-TCTGCCAGTGCCTCTTTGCT-3′

### Data analysis and statistics

Data analysis and graphing was performed in Microsoft Office EXCEL 2007 and GraphPad Prism 4.0c. The class membership analysis [16] was performed at Statistika Forskningsgruppen Stockholm, Sweden using STATA® (StataCorp LP, Texas, USA). All statistical analyses were performed in a 95% confidence interval.

### Ethics statement

Blood samples from 9 steroid resistant UC patients and 9 healthy volunteers were obtained with ethical approval from EPN (Regionala Etiska Prövningsnämnden, Stockholm, Sweden numbers 2005/1351-31/4 and 2005/1183-31/4 respectively).

The phase IIa study (EudraCT number: 2006-001846-15) was reviewed and approved by regional Independent Ethics Committees (IECs) and by the medical authorities in each country prior to inclusion of patients.

Named patient basis treatment was performed under the responsibility of the treating physician and with approval of the local ethic committee (Ethik-Komission der Ärztekammer Westfalen-Lippe und der Medizinischen Fakultät der Westfälischen Wilhelms-Universität Műnster, reference number 2008-360-f-S).

All patients had received written and verbal information concerning the study/treatment and signed an informed consent. From the named patient basis treated patient an additional written consent was obtained allowing the publication of individual clinical data.

## Results

### DIMS0150 induces cytokines having a role in steroid sensitivity

**Figure 1:**
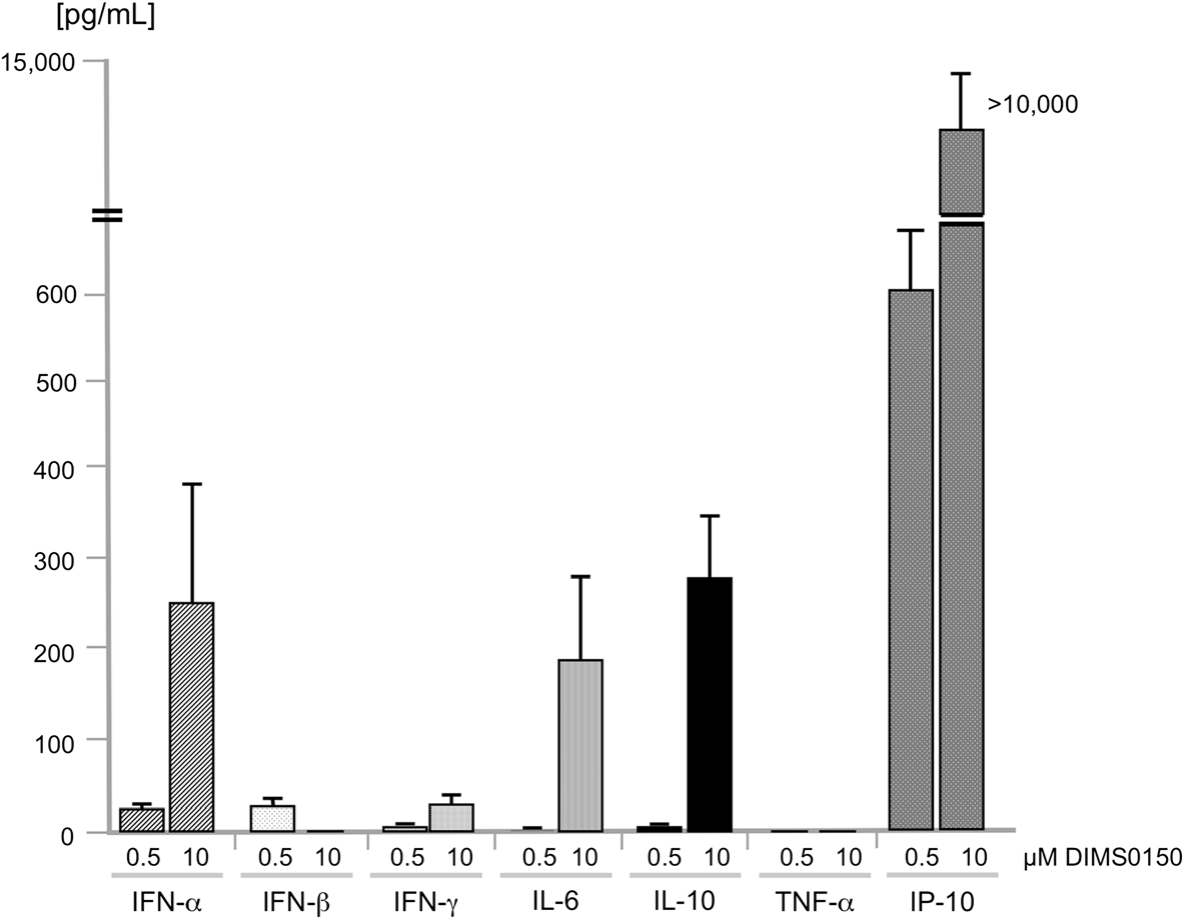
**Cytokine production by human PBMCs stimulated with DIMS0150.** Human PBMC from healthy volunteers (n = 5) were stimulated with 0.5 μM or 10 μM of DIMS0150 for 48 hrs. Cell culture supernatants were collected and analysed using CBA flex (IFN-γ, IL-6, IL-10, IP-10, and TNF-α) and ELISA (IFN-α and IFN-β). As control, cytokine levels obtained from PBMC incubated with medium alone were subtracted from all the values. Protein levels [pg/ml] are presented as mean values and SEM bars.

DIMS0150 is a fully synthetic oligodeoxynucleotide and its mode of action is thought to be triggered by the interaction with TLR9, which stimulates the production of specific chemokines and cytokines from mucosal immune cells, such as B-cells, macrophages and pDCs. To determine which cytokines are produced, *in vitro* studies were performed on PBMCs isolated from healthy subjects and stimulated with DIMS0150. The results demonstrated a pronounced production of anti-inflammatory cytokines like IL-10 and type I interferons and the chemokine IP-10 (Figure 1). Interestingly, both IL-10 and IFNs have been previously described as being able to modulate the steroid sensitivity of specific cells and thereby improved the response to steroids in steroid resistant individuals [7,8,17,18].

### Steroid response genes show steroid enhancing effect of DIMS0150

**Figure 2:**
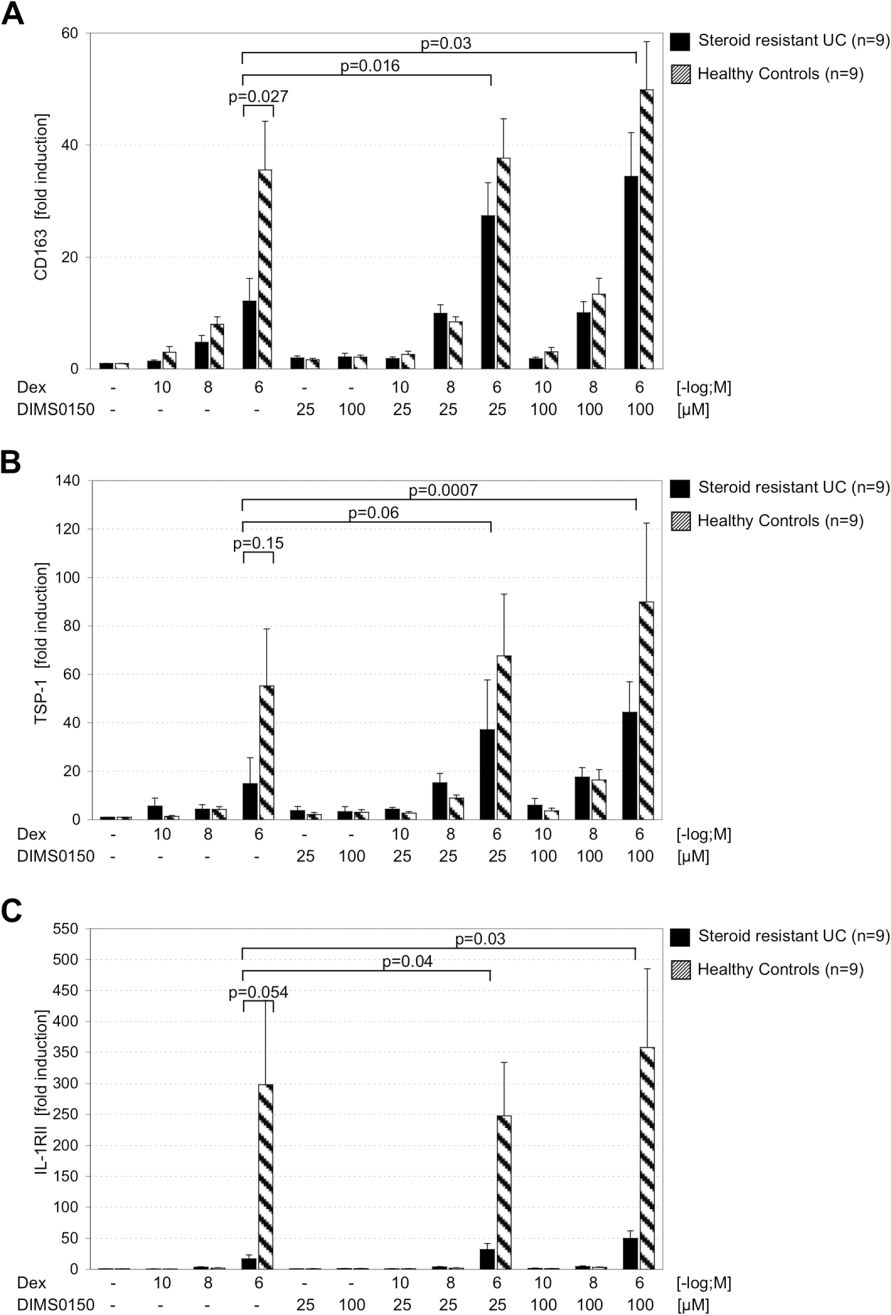
**DIMS0150 can enhance *in vitro *steroid sensitivity in steroid resistant UC patients.** PBMCs from 9 steroid resistant UC patients and from 9 healthy volunteers were incubated for 48 hrs in the absence or presence of Dexamethasone and/or DIMS0150 and the expression levels analysed for **(A)** CD163, **(B)** TSP-1 and **(C)** IL-1RII. The P-values between two groups were calculated using unpaired t-test, the P-values inside a group were calculated using paired t-test.

Through the induction of these steroid sensitizing cytokines by DIMS0150, it was speculated that DIMS0150 can sensitize to steroids and therefore was beneficial in the first pilot study in steroid resistant UC patients on concomitant steroid dosage. In order to investigate if DIMS0150 has indeed the ability to increase the steroid sensitivity of a patient, it was necessary to first identify suitable steroid response biomarkers. For this purpose blood samples derived from steroid resistant UC patients (n = 9) were obtained and a panel of 34 potential steroid response genes [4,17,19-32] were screened in comparison to healthy individuals (n = 9) (Table 3). As described in Galon et al., isolated PBMCs from steroid resistant patients and healthy volunteers were stimulated with three doses of Dexamethasone (10^-10^ M, 10^-8^ M, 10^-6^ M) and incubated with or without DIMS0150 (25 μM and 100 μM). To be selected as a potential biomarker for the sensitizing effect of DIMS0150, four criteria had to be fulfilled. Firstly, the individual variation in the expression of a gene in healthy volunteers should be minimal. Secondly, the effect of Dexamethasone stimulation should produce a reliable induction or repression. Thirdly, to discriminate between steroid resistance and normal sensitivity, a biomarker must demonstrate an impaired steroid response in the resistant group compared to healthy. Lastly, the biomarker should show a clear synergistic enhancement after co-stimulation with DIMS0150. Three genes (CD163, TSP-1 and IL-1RII) could be identified on the basis of their elevated expression levels when stimulated with DIMS0150 and Dexamethasone (Figure 2). When PBMCs derived from steroid resistant UC patients were stimulated with Dexamethasone, the level of CD163 induction was significantly lower compared to healthy controls suggesting a reduced level of steroid sensitivity. However, addition of DIMS0150 to the Dexamethasone treatment produced a significant enhancement of CD163 mRNA levels suggesting that DIMS0150 had restored the sensitivity of the PBMCs to Dexamethasone to a level approaching that of healthy controls (Figure 2A). By contrast, treatment of PMBCs with DIMS0150 alone had no effect. A similar relationship could be demonstrated for TSP-1 (Figure 2B) and Il-1RII (Figure 2C) with a clear reduced response to the applied Dexamethasone that could be significantly reversed through the addition of DIMS0150. Collectively, these three genes imply that DIMS0150 has steroid re-sensitizing activity and could potentially be used as biomarkers to identify subjects of reduced steroid sensitivity. Perhaps more importantly, they could also function as potential surrogate markers of a clinical response allowing identification of those subjects most likely to respond to DIMS0150 treatment.

**Table 3:** List of steroid response genes screened in biomarker assay

**Gene**	**Name**	**Gene ID**	**Reference**	**Sreening result**
TLR9	Toll-like receptor 9	54106	19, 20	3
TSP-1	Thrombospondin-1	7057	19	5
TSP-2	Thrombospondin-2	7058	19	1
IDO	Indoleamine 2,3-dioxygenase 1	3620	19	4
MARCO	Macrophage receptor with collagenous structure	8685	19	4
IL-1β	Interleukin 1, beta	3553	19	1
IL-1RI	Interleukin-1 receptor type 1	3554	19	4
IL-1RII	Interleukin-1 receptor type 2	7850	19, 21	5
IL-1Rα	Interleukin-1 receptor antagonist	3557	19	3
IL-1RAP	IL-1 receptor accessory protein	3556	20	3
IL-7Rα	Interleukin 7 receptor, alpha	3575	19	1
IL-13Rα2	Interleukin-13 receptor subunit, alpha 2	3598	19	1
CD163	Antigen CD163	9332	19	5
GZMA	Granzyme 1, serine esterase 3	3001	19	4
GILZ	Glucocorticoid-induced leucine zipper	1831	19	1
CD49D	Antigen CD49D; integrin alpha 4	3676	19	1
GRα	Glucocorticoid nuclear receptor, isoform alpha	2908	5, 20	3
GRβ	Glucocorticoid nuclear receptor, isoform beta	2908	19, 20	3
GRγ	Glucocorticoid nuclear receptor, isoform gamma	2908	20	3
HSP90α	Heat shock protein HSP 90, alpha	3320	22	2
HSP90β	Heat shock protein HSP 90, beta	3326	22	2
UGT2	UDP glucuronosyltransferase 2	7362	23	2
IRS-1	Insulin receptor substrate 1	3667	23	4
FoxP3	Forkhead box protein P3	50943	24	4
CFL1	Cofilin 1	1072	25	1
HDAC2	Histone deacetylase 2	3066	26	1
IGFBP2	Insulin-like growth factor binding protein 2	3485	27	1
MDR1	ATP-binding cassette, sub-family B (MDR/TAP), member 1	5243	4	1
VitD3R	Vitamin D (1,25- dihydroxyvitamin D3) receptor	7421	17	1
FKBP51	FK506 binding protein 5	2289	28	3
GROα	Growth-regulated gene-alpha	2919	29	3
VEGFA	Vascular endothelial growth factor	7422	30	2
SGK	Serum- and glucocorticoid-inducible protein kinase	6446	31	1
IFNγR1	Interferon gamma receptor 1	3459	32	1

(1)Excluded because of too high individual variability in healthy volunteers.

(2)Excluded because Dexamethasone (Dex) response in healthy volunteers was not reliable.

(3)Excluded because difference in Dex response between steroid resistant patients and healthy volunteers was not reliable.

(4)Excluded because of missing or not reliable synergistic effect when stimulated with Dex and DIMS0150.

(5)Selected as potential biomarker because all four criteria were fulfilled (see text).

### DIMS0150 provides clinical benefit in patients with UC on concomitant steroid therapy

**Figure 3:**
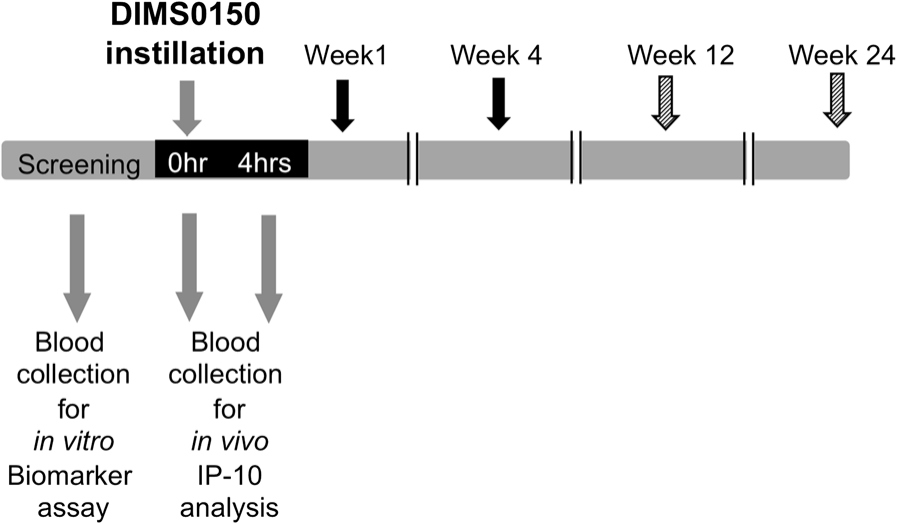
**Design of the explorative phase IIa study.** Steroid resistant and steroid dependent UC patients were included in a placebo controlled double-blind study to re-affirm the target group for the treatment with DIMS0150 and for evaluation of biomarkers. (Black arrow) efficacy measure time point, (dashed arrow) follow-up time point.

To investigate the clinical usefulness of these steroid sensitivity biomarkers as surrogate markers for DIMS0150, a PoC phase IIa study conducted to confirm the target group of DIMS0150 was used. The objective of this study was to evaluate the clinical response following single dose treatment with DIMS0150 compared to placebo in steroid resistant or steroid dependent UC patients on concomitant steroid medication. Clinical responders were defined as patients showing a decrease in the Disease Activity Index (DAI) [2] score of ≥3 points from baseline and the difference in the number of responders in the two groups (active versus placebo) was assessed at two different time points with two further follow-up visits to gain information on next relapses and the safety profile (Figure 3). The results from this study confirmed the efficacy observations made in the first clinical study in that a notable improvement of clinical symptoms was observed one week after a single dose of DIMS0150 (Table 4). Additionally, it could also be shown that only those patients that had a clinical response following DIMS0150 treatment experienced a sustained clinical response or remission and that clinical remission was paralleled with mucosal healing (histological remission [33]). DIMS0150 demonstrated a very favourable safety profile with no significant differences in adverse events between the treatment and placebo groups.

**Table 4:** Summary of the efficacy data proof of concept phase IIa study

	**ITT population (n = 34)**		
	**Placebo (n = 12)**	**DIMS0150 (n = 22)**	**P value**
**Clinical response**^**1**^			
Week 1 (wk1)	2 (17%)	7 (32%)	0.43
Week 4 (wk4)	4 (33%)	9 (41%)	0.71
**Sustained clinical response:**			
Wk1 and wk4	0/12 (0%)	6/22 (27%)	0.06
**Clinical remission**^**2**^			
Wk1	1 (8%)	2 (9%)	1.0
Wk4	0 (0%)	3 (13%)	0.27
**Sustained clinical remission:**			
Wk1 and wk4	0/12 (0%)	2/22 (9%)	0.18
**Histological response**^**3**^			
Wk4	0 (0%)	6 (27%)	0.06
**Sustained histological response:**			
Wk4 and wk12	0/8 (0%)	3/16 (19%)	0.2
**Histological remission**^**4**^			
Wk4	0 (0%)	6 (27%)	0.06
**Sustained histological remission:**			
Wk4 and wk12	0/8 (0%)	3/16 (19%)	0.2
**Clinical response paralleled with histological remission**			
Wk4	0/4 (0%)	4/9 (44%)	0.22

^**1**^defined as DAI score decrease of at least 3 points from baseline.

^**2**^defined as a total DAI score of 2 points or lower, with no individual sub-score exceeding 1 point.

^**3**^defined as histology score decrease of at least 3 points from baseline.

^**4**^defined as histology score decrease to a score of zero.

No wk24 data of patients with sustained remission available.

(ITT) Intention-To-Treat; P values calculated with Fisher’s Exact Test for response/remission rates.

### Evidence of TLR9 activation by DIMS0150

**Figure 4:**
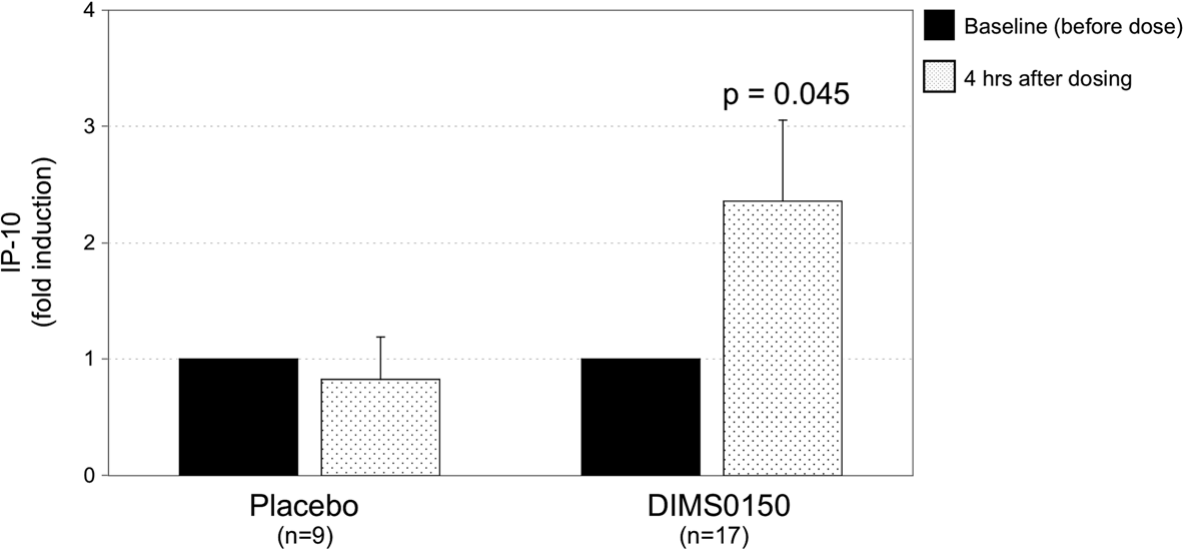
**Pharmacological activity of DIMS0150.** In the explorative phase IIa study, whole blood was collected before and 4 hrs after DIMS0150 treatment and the levels of IP-10 mRNA measured through qPCR. The P-value was calculated between placebo and DIMS0150 treated group using unpaired t-test.

Interferon-γ induced protein 10 (IP-10) is known to be a sensitive *in vivo* marker for the pharmacological activity of CpG containing oligonucleotides that act through the TLR9 pathway [34-36]. As DIMS0150 is capable of inducing IP-10 *in vitro* (see Figure 1), we set out to measure IP-10 *in vivo* as evidence of the TLR9 stimulation. Whole blood samples from 26 patients were obtained in order to monitor the expression of IP-10 mRNA (see Figure 3). Once the clinical study had been un-blinded, the IP-10 expression data were compared in terms of whom received study drug or placebo. The qPCR analysis indicated that IP-10 expression was significantly increased in the DIMS0150 treatment group 4 hrs after dosing, whereas no change in IP-10 expression was observed in the placebo group (Figure 4).

### Biomarkers identify two groups of differing steroid sensitivity

Blood derived PBMCs were isolated from 32 patients from the PoC phase IIa study at the time of screening, treated *in vitro* with Dexamethasone and/or DIMS0150 for 48 hrs and the expression levels of CD163, TSP-1, IL-1RII and IL-6 determined through qPCR analysis. In response to stimuli, levels of IL-6 are increased and the degree of suppression by GCS is indicative of the steroid sensitivity. Indeed, the ability of GCS to suppress the levels of induced IL-6 appears to be an accepted measure of steroid sensitivity as there appears to be a robust correlation with the clinical picture of steroid resistance [11,13,14]. The use of IL-6 circumvents the need for patients to undergo what are otherwise uncomfortable treatments such as taking high levels of steroid i.v. over a period of 5 days to determine the subjects’ steroid sensitivity.

**Figure 5:**
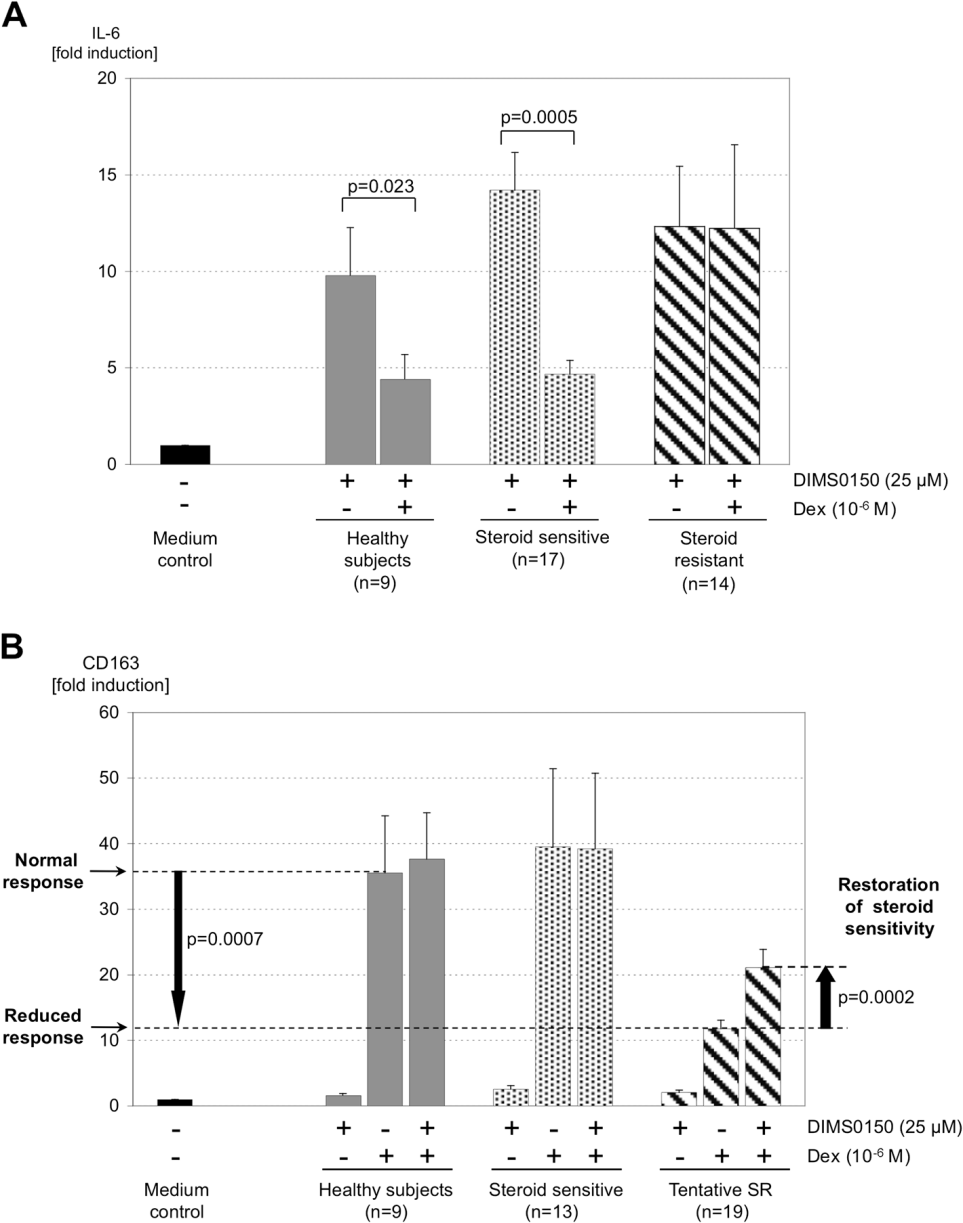
**Biomarkers detect two subgroups of patients in the phase IIa study.** Whole blood was collected at screening from 32 patients and PBMC incubated for 48 hrs in the absence or presence of Dexamethasone and/or DIMS0150. The expression levels for **(A)** IL-6 and **(B)** CD163 were analysed and the data divided into two groups using arbitrary cut-offs. In case of IL-6, one patient had an outlying IL-6 stimulation (>100 fold) and was removed from the analysis. The P-values between two groups were calculated using unpaired t-test, the P-values inside a group were calculated using paired t-test.

Prior to un-blinding, two types of responses in the IL-6 biomarker assay were observed (Figure 5A). One group appeared to behave like healthy controls demonstrating a strong reduction of the induced levels of IL-6 following Dexamethasone incubation (steroid sensitive patients), whereas no suppression of IL-6 was noted in the other group (steroid resistant patients). Also with the biomarkers CD163, TSP-1 and IL-1RII, two differing groups of steroid responses could be observed as shown by the example of CD163 (Figure 5B). One group appeared to behave like healthy controls when stimulated with Dexamethasone or Dexamethasone combined with DIMS0150 suggesting that these patients had a comparable steroid sensitivity. By contrast, the other group had a significant reduction in the level of Dexamethasone-induced CD163 expression compared to healthy controls. Furthermore, this reduced response could be significantly enhanced by the addition of DIMS0150 confirming previous *in vitro* observations that DIMS0150 is able to restore steroid sensitivity in steroid resistant patients suggesting that the patient group with reduced steroid response are steroid refractory.

Both biomarkers, IL-6 and CD163, were able to identify the same two groups, namely patients being resistant to steroids and those being similar to healthy controls and segregate between steroid refractory and sensitive patients. TSP-1 as well as IL-1RII could also segregate between two differing steroid sensitivity groups whereby the correlation between IL-6 and CD163 or TSP-1 was in both cases over 71% and IL-1RII showed only a correlation of about 45% (data not shown).

### Biomarkers have high potential in predicting clinical response to DIMS0150

Upon PoC phase IIa completion, we determined whether there was a correlation between patients showing a reduced steroid sensitivity in the biomarker assay before treatment and clinical response. In other words, is steroid resistance a pre-requisite for a response to DIMS0150 and could this be used as a predictor. Of equal interest was to determine whether those subjects that had previously demonstrated in the biomarker assay that DIMS0150 could restore their *in vitro* steroid sensitivity had an actual clinical response to DIMS0150.

For this purpose, classification analyses were performed using the raw data from the relative quantification analysis of the performed *in vitro* assay compared to the clinical outcome. From the 22 patients receiving DIMS0150, 10 patients had a clinical response at week 1 and/or week 4 and 12 patients had no clinical response either at week 1 or at week 4. For all the statistical analysis the first group of patients were classified as clinical responders and the second group as clinical non-responders. Table 5 provides values for area under the ROC (Receiver operating characteristics) curves (AUC) when using each of the markers CD163, TSP-1 and IL-6 separately and in combination at two concentrations of Dexamethasone (10^-8^ and 10^-6^ M) and 25 μM DIMS0150.

**Table 5:** Area under the ROC curve (AUC) calculations for efficacy prediction of the biomarkers

		**CD163**	**TSP1**	**IL-6**	**CD163/TSP-1/IL-6**
Steroid response	Dex 10^-8^	0.86	0.88	0.60	0.91
	Dex 10^-6^	0.85	0.87	0.83	0.91
Steroid enhancement	Dex 10^-8^	0.82	0.80	N/A^#^	0.86*
	Dex 10^-6^	0.77	0.81	N/A^#^	0.96*
Steroid response and enhancement	Dex 10^-6^	-	-	-	**0.98**

^#^Not applicable as the level of repression of DIMS0150 induced expression of IL-6 is analyzed.

*Calculation was performed including steroid response data of IL-6.

**Bold number** indicates highest efficacy prediction.

The results indicated that the three biomarkers, CD163, TSP-1 and IL-6, had singularly high predictive potential for a clinical response in subjects that demonstrated reduced levels of steroid sensitivity suggesting that refractory patients are more likely to respond to DIMS0150. Additionally, CD163 and TSP-1 demonstrated a strong positive correlation between subjects where a restored *in vitro* steroid sensitivity was observed upon DIMS0150 incubation and clinical response. IL-1RII had less discriminative potential due to strong patient variations (data not shown). However, the highest prediction values were achieved when all three biomarkers CD163, TSP-1 and IL-6 were considered for steroid resistance and, taking into account restoration of steroid sensitivity as determined by CD163 and TSP-1. This gave an AUC of 0.98 with 10^-6^ M Dexamethasone and 25 μM DIMS0150 in contrast to an AUC of 0.83 for IL-6 alone at the same concentrations (Table 5, see also Figure 5A).

**Figure 6:**
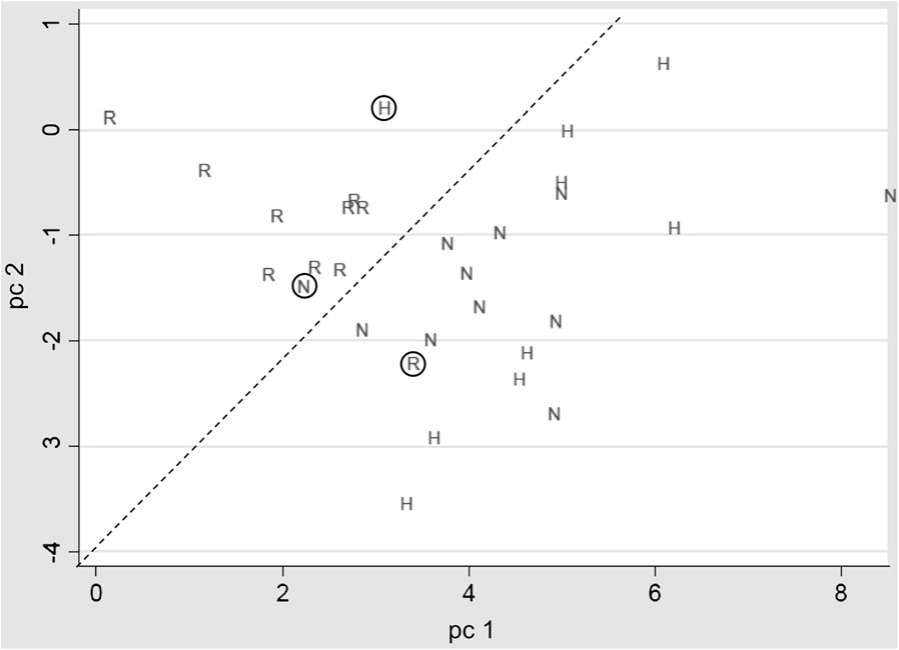
**Classification analysis confirmed clinical responding and non-responding patients as two distinct groups.** Principal components (pc) were calculated from the logarithms of the steroid response data received from the qPCR analyses of CD163, TSP-1 and IL-6 and pc1 against pc2 was plotted. (R) Clinical responder. (N) Clinical non-responder. (H) Healthy volunteer. Circles present subjects whose pc data are differing from their respective group.

For graphical illustration, the principal components (pc) were calculated from data derived from CD163, TSP-1 and IL-6 and the pc1/pc2 distribution plotted, reflecting the differences in the gene expression of these three genes in responding and non-responding patients (Figure 6). The healthy data points co-located with data points from the non-responders suggesting that both groups had a normal level of steroid sensitivity.

Table 6 shows the level of agreement between the prediction of a clinical response and the actual clinical response for each patient receiving the study drug. Considering either the criteria for steroid resistance as determined by the biomarkers or whether stimulation with DIMS0150 was able to reverse the steroid resistance in the assay, a correct prediction of 81% (17/21) was obtained (Table 7). When using the combination of both criteria namely, steroid resistance and enhancing steroid sensitivity an overall correct prediction of 90.5% (19/21) with a positive predictive value (PPV) of 90% and a negative predictive value (NPV) of 91% was achieved. Only two patients did not correlate with the biomarker prediction, patient N08 who showed a positive result in the biomarker analysis but no clinical response and patient R05 who was negative in the biomarker analysis but experienced a clinical response. Two further patients, N04 and N05, received study drug but were not on concomitant steroid treatment. They were later excluded from the study as protocol violators. Both of them were classified as being steroid sensitive in the biomarker assay and were clinical non-responders.

**Table 6:** Prediction of clinical response in DIMS0150 treated patients (n = 22) using CD163, TSP-1 and IL-6

**Patient**	**Steroid response**	**Enhancing**	**Steroid response and enhancing**
N01	1	0	0
N02	0	0	0
N03	0	0	0
N04	0	0	0
N05	0	0	0
N06	0	1	0
N07	0	0	0
N08	1	1	1
N09	0	0	0
N10	0	0	0
N11	0	0	0
N12	n.d.	n.d.	n.d.
R01	1	1	1
R02	1	0	1
R03	1	1	1
R04	1	1	1
R05	0	0	0
R06	1	1	1
R07	1	1	1
R08	1	1	1
R09	0	1	1
R10	1	1	1

(N) Clinical Non-responder defined as DIMS0150 treated patients with no clinical response at week 1 and week 4.

(R) Clinical Responder defined as DIMS0150 treated patients with a clinical response at week 1 and/or 4).

(0) Negative in classification analysis (i.e., steroid sensitive and/or not enhanced).

(1) Positive in classification analysis (i.e., steroid resistant and/or enhanced).

(n.d.) No data (no blood was received from patient for biomarker analysis).

**Table 7:** Summary of predictive potential of biomarkers CD163, TSP-1 and IL-6

	**Correct prediction**	**Sensitivity**	**Specificity**	**PPV**	**NPV**
**Steroid response**	81% (17/21)	80%	82%	80%	82%
**Enhancing**	81% (17/21)	80%	82%	80%	82%
**Steroid response and enhancing**	90.5% (19/21)	90%	91%	90%	91%

(PPV) Positive predictive value.

(NPV) Negative predictive value.

To determine whether using these biomarkers as a stratifying tool may have improved the clinical response rate, we performed a retrospective analysis using the study data. By removing all subjects that were neither steroid resistant nor showed an enhancing effect to DIMS0150 in the biomarker assay and retaining all those that proved positive in both criteria, the response rate in the treatment group would have increased from 32% to 60% (6/10) at wk1 and from 41% to 80% (8/10) at wk4. These data demonstrate a clear usefulness in being able to identify patients most likely to benefit from DIMS0150 treatment.

### DIMS0150 restores steroid sensitivity *in vivo*

**Figure 7:**
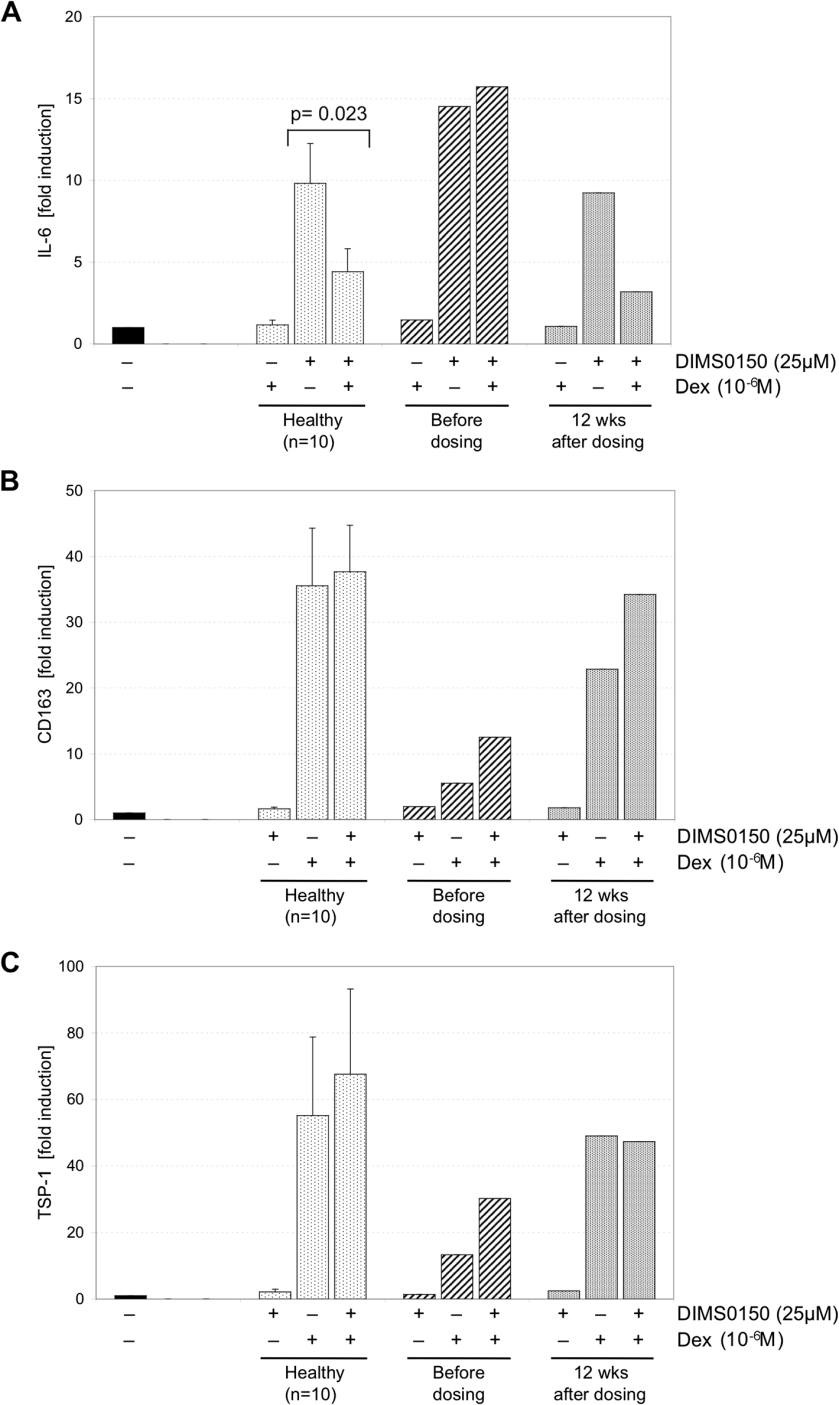
**Biomarker assay confirms restoration of steroid sensitivity.** A chronic active treatment refractory UC patient was treated on a named patient basis with three doses of DIMS0150 with four weeks between dosing. Whole blood was collected prior to first dose and four weeks following last dose (week 12) and PBMC incubated for 48 hrs in the absence or presence of Dexamethasone and/or DIMS0150. Following incubation mRNA levels of IL-6 **(A)**, CD163 **(B)** and TSP-1 **(C)** were analysed.

In a case series of severe chronic active treatment refractory patients, treatment with DIMS0150 was provided under named patient basis [15]. Here we had the opportunity to observe the effects of DIMS0150 treatment in a subject receiving three doses with four weeks between each dosing occasion. We noted an impressive response with the subject in complete clinical remission at week 12 (defined as a CAI score of 0 and an endoscopic score of 0) [37], 4 weeks after the third dose. Additionally, we were able to collect blood before dosing and 12 weeks after for the analysis of CD163, TSP-1 and IL-6 (Figure 7). The analysis showed a strong reduced steroid response indicating that the patient was steroid refractory at the time of first dosing which is supported by the subject’s treatment history of being resistant to a high course treatment consisting of 100 mg Prednisolone/day for 5 days. After three administrations of DIMS0150, patient derived PBMCs responded similar to healthy controls when incubated with Dexamethasone. This would suggest that a restoration of sensitivity must have occurred *in vivo* during DIMS0150 treatment. After a further 14 weeks the patient experienced first disease deteriorations (increase of the CAI score from 0 to 4) and in light of the subject’s apparent improved steroid sensitivity, the treating physician was advised to increase the dose of GCS from 10 to 30 mg of Prednisolone/day. The patient regained complete remission within a week suggesting that following a period of re-sensitization to GCS, the patient was able to respond to the elevated dose given.

## Discussion

Gastrointestinal disorders like UC can dramatically affect the quality of life [38], and involves a life-long clinical management of the disease focusing on the induction and maintenance of remission. GCS remain the treatment of choice of initial therapy but about a third of the patients will fail to respond and further management requires a comprehensive understanding of the patients and the potential risks and benefits of further interventions making the disease course difficult to manage. The aim of the treatment with DIMS0150 is to help the patients remain in the group of easier manageable UC patients by restoring their steroid sensitivity.

The mechanisms behind steroid resistance are complex and numerous cytokines have been implicated as important factors. For example, Xystrakis and colleagues^. ^[18] could show that by restoring otherwise deficient levels of IL-10 in steroid resistant asthmatics greatly improved their steroid responsiveness. In a more recent study performed with PBMCs derived from steroid resistant UC patients, the authors could demonstrate that addition of IL-10 to the PMBCs enhanced steroid sensitivity, whereas neutralizing IL-10 through addition of specific antibodies reduced steroid sensitivity [8].

Similar clinical observations have been demonstrated using type I interferons. For example, steroid resistant UC patients receiving daily intravenous injections of natural IFN-β experienced a rapid improvement of clinical symptoms [39]. The ability of type I interferons to modulate steroid sensitivity gained further support from studies performed in steroid resistant asthmatics where treatment with IFN-α dramatically improved symptoms allowing their steroid dose to be tapered [19].

We could show that the TLR9 agonist DIMS0150 acts as an immunomodulatory compound by inducing IL-10, IL-6, type I interferons and IP-10 *in vitro*, the same cytokines/chemokines implied to be important in regaining steroid sensitivity. The induction of IP-10 *in vivo* in the phase IIa study correlates well with the *in vitro* IP-10 analysis suggesting that treatment with DIMS0150 is likely to induce the same steroid enhancing cytokines as previously recorded.

The biomarker CD163 belongs to a superfamily of cysteine-rich scavenger receptors (SRCR), several of which are involved in the innate immune response [40]. CD163 is described to mediate anti-inflammatory effects [41] and its expression is strongly induced by anti-inflammatory mediators and GCS [20,42]. Interestingly, some of these anti-inflammatory mediators that up-regulate CD163 on mRNA level in monocytes and macrophages are IL-6 and IL-10 [43-45], both of which are shown to be induced by DIMS0150.

Thrombospondin-1 belongs functionally to a group of diverse multidomain counteradhesive proteins influencing endothelial cell behaviour [46-48]. It could be shown that TSP-1 expression correlates with IL-10 expression in colon cancer with significant lower mean vessel counts suggesting that IL-10 stimulates expression of angiostatic factors as TSP-1 [49], linking also the second biomarker to a cytokine induced by DIMS0150. The identification of these marker genes and their relation to DIMS0150 induced cytokines are considered important factors in understanding how DIMS0150 restores steroid sensitivity.

To assess the clinical utility of CD163, TSP-1 and IL-1RII as predictors of clinical response, a PoC phase IIa study in steroid refractory or steroid dependent UC patients on concomitant steroid treatment was performed and the results compared to those of the steroid sensitivity marker IL-6. Although this study was somewhat limited in size, it was nevertheless deemed sufficiently large enough to provide a robust assessment of the biomarkers. We hypothesized that DIMS0150 should enhance steroid sensitivity leading to improvements of symptoms and a reduced disease activity score and that prior analysis using the biomarker genes should enable a prediction of clinical response. The clinical outcome of the study showed that approximately half of the DIMS0150 treated patients (10 of 22) responded to the treatment and that this observation was very much in line with the biomarker data obtained at the time of screening. Both IL-6 and CD163 analysis strongly suggested the presence of two groups of patients being included in the study. One group demonstrated a clear picture of steroid resistance and the other showed a steroid response similar to healthy volunteers. Patients with a reduced response to steroids as determined by CD163, TSP-1 or IL-6 were statistically more likely to respond to the DIMS0150 treatment. The predictive potential of the biomarkers could be illustrated by classification analysis of the expression data compared to clinical response or non-response following DIMS0150 treatment. All three markers demonstrated a high potential as surrogate markers for a DIMS0150 response with CD163 and TSP-1 being slightly more sensitive than IL-6 because of their ability to demonstrate the steroid re-sensitizing effect of DIMS0150. As expected, the combination of all three markers gave the best result with an AUC of 0.98. This equates to a correct prediction of clinical response in 90% (9/10) of patients classified as being steroid refractory according to the biomarker assay. Conversely, 91% (10/11) of patients whose steroid sensitivity was comparable to healthy controls failed to respond to DIMS0150 treatment. A possible interpretation would be, patients classified with the biomarkers as steroid refractory are indeed steroid refractory patients. By contrast, patients that show no difference to healthy could be inferred as steroid dependent. The validity of these interpretations can only be properly tested through additional clinical studies where subjects are included using the clinical definitions of steroid resistance and dependence as given in the ECCO guidelines [10].

Regarding the special case patient who received three doses of DIMS0150, the biomarker assay confirmed that the patient was steroid refractory at time of first dosing and classified as a potential responder to DIMS0150. Upon treatment with DIMS0150, a pronounced clinical response could be observed with the patient in complete remission at week 12. Further biomarker analysis at week 12 demonstrated that the patient most likely had regained steroid sensitivity that could be confirmed by treating an upcoming relapse successfully with GCS. While we have consistently recorded a steroid re-sensitizing effect for DIMS0150 *in vitro*, these *in vivo* data provide for the first time, evidence for a shift to improved steroid sensitivity in a steroid unresponsive patient following DIMS0150 treatment.

Based on these promising data, a placebo-controlled, multiple dose, double-blind, randomized phase III clinical study (NCT01493960) is currently on-going to assess the efficacy and safety of DIMS0150 as an add-on to current practice in chronic active treatment refractory UC patients.

This study will also provide a unique opportunity to gain further evidence for the observed *in vivo* shift in steroid sensitivity following DIMS0150 treatment and controlled steroid tapering combined with a long follow-up phase will gather information about reaching and length of steroid-free remission.

## Conclusions

The work presented here demonstrates that the target group of DIMS0150 are ulcerative colitis patients showing a reduced steroid sensitivity and a clear utility of using appropriate biomarkers for the selection of patients most likely to benefit from DIMS0150 treatment has been illustrated. Using such an approach represents a step towards a more personalized form of healthcare and may aid physicians in making the most optimum treatment choices for the patient.

## Competing interests

Nikolai V. Kouznetsov, Arezou Zargari, Alexander Gielen, Oliver von Stein and Petra von Stein received salaries from InDex Pharmaceuticals. Nikolai V. Kouznetsov, Arezou Zargari, Alexander Gielen and Petra von Stein have equity interest in Index Pharmaceuticals; Oliver D. von Stein and Robert Lofberg have stock ownership at Index Pharmaceuticals.

## Authors’ contributions

PvS had substantial contribution in conception and design of work, data collection, data analysis and interpretation, drafting of manuscript; NK had substantial contribution in biomarker selection and screening, data collection, data analysis and drafting the manuscript; OvS participated substantially in conception and design of work, interpretation of data and critically revising the manuscript; RL was substantially involved in study design (blood collection studies and PoC study), acquisition of data and critical revision of manuscript; RB was substantially involved in study design (PoC study), acquisition of data and critical revision of manuscript; EM had substantial contribution in treatment of patient under name-patient basis (work design and acquisition of data) and critical revision of the manuscript; AZ and AWG were both substantially involved in data collection, data analysis and critical revision of the manuscript. All authors read and approved the final manuscript.

## Pre-publication history

The pre-publication history for this paper can be accessed here:

http://www.biomedcentral.com/1471-230X/14/79/prepub
